# Associative Visual Agnosia: A Case Study

**DOI:** 10.1155/2008/241753

**Published:** 2008-04-11

**Authors:** A. Charnallet, S. Carbonnel, D. David, O. Moreaud

**Affiliations:** ^1^CMRR & NeuropsychologiePôle de Psychiatrie et NeurologieCHU de GrenobleFrance; ^2^Laboratoire de psychologie et neuro-cognition(CNRS UMR 5105)Université de SavoieChambéry & Université Pierre Mendes FranceGrenobleFrance

**Keywords:** Associative visual agnosia, semantic memory, visual knowledge, episodic memory

## Abstract

We report a case of massive associative visual agnosia. In the light of current theories of identification and semantic knowledge organization, a deficit involving both levels of structural description system and visual semantics must be assumed to explain the case. We suggest, in line with a previous case study [1], an alternative account in the framework of (non abstractive) episodic models of memory [4].

## 1. Introduction

Object identification is usually viewed as requiring access to stored semantic representations, following several sequential processing stages (perceptual processing, access to stored structural descriptions, access to semantic knowledge). This view has constituted a useful theoretical framework to assess and explain most cases of patients presenting with visual agnosia or semantic deficits. Unfortunately, some cases are not easily accounted for by reference to this conceptual approach. Such a case (patient EC) has been previously described by Carbonnel et al. (1997) [1] and interpreted in a quite different framework, the non abstractive view, assuming the episodic nature of knowledge and proposing that episodic and semantic behaviors arise from the same system of memory traces [4,5]. The present paper reports the study of an agnosic patient presenting with a pattern of deficits similar to EC's case.

## 2. Case study

### 2.1. Clinical history

JPM (born 1948) is a right-handed man, with high educational level. He presents with massive visual agnosia and complete alexia ten months after cerebral anoxia secondary to cardiac arrest. He also exhibits a frontal lobe syndrome with mild memory impairment, a severe constructional apraxia and deficits in execution of symbolic gestures and in pantomime of object use on verbal command. His language is fluent, informative, but with mild word finding difficulties and numerous paraphasias in spontaneous speech. Neurological and visual field examination, and brain MRI are normal.

### 2.2. Object recognition

Object identification (assessed by naming and/or definitions) is severely impaired and involves all categories of visual stimuli: JMP succeeds in recognizing only 7/48 photographs of usual inanimate objects, 3/48 "living items" (animals and plants), 4/25 famous faces and 3/22 famous buildings (*no significant difference between categories*).

His performance doesn't improve in forced choice recognition tasks (pointing among several the picture of a named object or deciding whether a picture represents a given name).

On the visual form of the PPTT [6] he scores 21/52, what is at chance level.

ISSN 0953-4180/08/$17.00 © 2008 – IOS Press and the authors. All rights reserved

### 2.3. *Visual perception*

Perceptual processing was assessed by several same/different judgment tasks, with various stimuli. The results of this investigation are summarized in Table 1.

JPM scores at a normal level, in each of the five tasks. Thus, it can be assumed that he doesn't present any obvious perceptual deficit and his visual agnosia can be characterized as a massive associative agnosia.

### 2.4. *Knowledge from non-visual input*

In contrast, access to semantic knowledge from other modalities is much better preserved (e.g. 48/52 on the verbal form of the PPTT). However, a category effect is observed. In sound identification, performance is at normal range, for nature sounds (e.g. thunder, sea waves. . . , 100%) and for artefacts (e.g. train, telephone ring. . . , 92%), whereas it falls below values obtained by normal control subjects for animals (e.g. dog barking, pig growling. . . , 72%).

From a verbal input, knowledge of structural (visual) and functional (non visual) properties was systematically assessed[1], both for animals ($n = 100$) and for artefacts ($n = 100$) using definitions from names and various property judgment tasks (e.g.: is a . . . dangerous? . . . has a . . . a handle?, see Appendix 1). Results for the two tasks put together are summarized in Table 2.

Compared to functional knowledge, knowledge of visual properties is clearly impaired, both for artefacts (Chi2 $= 21$, $p < 0.001$) and living items (Chi2 $=$ 4.7, $p = 0.03$), and to the same extent for these two categories (Chi2 $=$ 0.6, $p > 0.30$).

In contrast, results point out a dissociation between the two categories regarding non visual knowledge (Chi2 $=$ 13.2, $p < 0.001$): they are almost normal for artefacts and clearly impaired for living items (examples of JPM's definitions are presented in Appendix 2).

The massive deficit of visual knowledge was further evaluated, both for living items (animals) and artefacts, by 3 additional tasks tapping structural knowledge both from visual (object decision) and verbal (object imagery tasks, adapted from Mehta, Newcombe & De Haan, 1992 [9]) input.

In object decision task, JPM had to decide whether a picture portrays a real object (40 artefacts and 21 animals) or a "non object" (40 non objects and 21 chimerical animals). He scores at chance level, both for artefacts (52.5% correct) and for animals (54.7% correct).

In object imagery task, the names of 3 objects were presented and JPM was asked to choose the 2 most similar with respect to their outline shapes (e.g. axe, flag, broom). The same task was proposed with animal names, but in this case, only the shape of the heads had to be considered (e.g. tiger, dog, cat). There were 12 and 13 trials respectively.

His performance is at chance level in both tasks (38.4% and 33.3% correct).

These last results confirm JPM's lack of knowledge about visual characteristics of objects.

## 3. Discussion

In summary, JMP's case is characterized by 1) associative visual agnosia and severe degradation of visual knowledge, both involving all categories and 2) from a verbal input, deficit in non visual knowledge restricted to living items.

According to current semantic memory models (e.g. [2,7]) a deficit of stored structural descriptions would provide a clear-cut explanation of JPM's complete visual agnosia and also of his lack of visual knowledge. On the other hand, the category specific impairment from verbal input, could be accounted for by a further semantic deficit. SFT [3,13] or HIT [8] theories predict that an impairment of visual knowledge would lead to such a specific deficit for the category of living things.

Alternatively, the whole pattern of impairment is predicted by episodic (non abstractive) models of memory [4,5] by assuming a single causal deficit. According to these models, there are no semantic representations permanently stored in memory. The meaning of an entity momentarily emerges from the reactivation of previous episodes experienced with this entity. An episode is made of several "components" reflecting the activity of the various sensory modalities (visual, motor, auditory. . . ) engaged during the interaction with an object. Schematically, a retrieval cue (e.g. the picture of an object) will trigger the reactivation of all episodic traces in proportion of their similarity with this cue and the components shared by most activated traces (the "echo") will constitute the meaning currently evoked of the object. The other components, more rarely present in episodes, will not be part of this first echo and will be evoked only at a second stage, when the information

[1] JPM's severe constructional apraxia prevented the assessment of structural knowledge from drawing tasks

Table 1
JPM's performance in 5 perceptual tasks

| | | Same | Different | Score |
|---|---|---|---|---|
| *2D Shapes* | Thurstone's items |  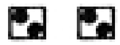 |  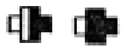 | 20/20 |
| | Blobs |  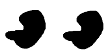 |  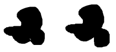 | 38/40 |
| | Objects |  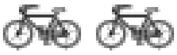 |  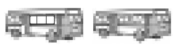 | 9/10 |
| | Non objects |  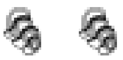 |  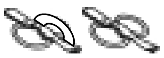 | 10/10 |
| *3D Shapes* | Greebles |  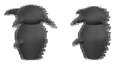 |  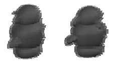 | 15/20 |

The subject was asked to judge if two stimuli were same or different, with 2D drawings (Thurstone's items [7], "blobs", objects and non objects) and 3D shapes (Greebles ; M.J Tarr, 2002)

Table 2
Percentage of correct responses for visual and functional (non visual) properties of artefacts and living items, assessed by definitions and property judgment tasks

| | Functional properties | Visual properties | |
|---|---|---|---|
| Artefacts (n=100) | **90.8%** | *66.6%* | |
| | s*** | s*** | ns |
| Living (n=100) | *71.7%* | *60%* | |
| | | s* | |

*ns : non significant difference*
*s* : significant at p<0.05*
*s*** : significant at p<0.01*

included in the echo will add to the retrieval cue, allowing to reactivate other traces, thus giving rise to a new and more complete meaning (re-injection process).

Within this theoretical framework JPM's picture can be explained by assuming a single functional disorder: the unavailability of the visual part of all episodic traces leading 1) to the lack of any visual information in the echo, whatever the cue and 2) the inability to reactivate any trace (and, thus, any meaning) from a visual retrieval cue, insofar as there will be no similarity between the traces and the cue. This accounts for the loss of visual knowledge and for visual agnosia.

To explain, in the same framework, the category specific impairment from verbal input, we assume, in line with Warrington and co-workers [12,13] that, given the conditions in which living vs. nonliving things are learned, most episodes involving objects comprise two invariant "components": the "visual" one and the "functional" one, whereas the visual component alone is part of most experiences with animals. It follows that, if the retrieval cue is a word, the functional component shared by the activated traces will become directly available in the echo for objects; for animals, the echo deprived of the visual component normally common to most episodes will fail to enhance the retrieval cue, thus preventing the re-injection process necessary to the evocation of non visual knowledge. A lack of this knowledge from animal names can thus be expected, whereas it will be still available from object names, as has been observed in JPM.

## Appendix 1: property judgment tasks

JMP was asked to give a yes/no answer for the following questions assessing visual and non visual properties of objects and animals.

| | Objects | Animals |
|---|---|---|
| Visual Properties | *– does a . . . have a handle?*<br>*– does a . . . have a grip?*<br>*– size (is a . . . smaller than a visible target object)?* | *– has a . . . horns?*<br>*– has a . . . legs?*<br>*– size (is a . . . smaller than a visible target object)?* |
| Functional properties | *– is a . . . used to contain something*<br>*– is a . . . used to warm up*<br>*– is a . . . used to carry something* | *– is a . . . eatable?*<br>*– is a . . . dangerous?*<br>*– does a . . . run faster than a man?* |

## Appendix 2: examples of JMP's definitions

Frog: *in the field of fishes, it lives in water. It's quite small (shows 1 cm), it lives in France besides the sea.*

On questions: *maybe eatable, I'm not sure; It has 2 legs; Has feathers; No idea of its sound*

Sheep: *a common animal, eatable, I believe, much smaller than a pig.*

On questions: *his usefulness?, to get around, it runs fast. . . what else can you do with a sheep?; Has horns; very long hairs on the body; The ears? I don't know; What sound can it make? I really don't know*

Binoculars: *in the field of vision tools. It is used to enlarge what you see, to see by far.*

On questions: *I don't know what it looks like, 3 parts, a rather round shape and you turn it . . . I'm in descriptive distress!*

Hammer: *a tool, very hard, which is used for the woodwork and do-it-yourself, to push in nails. Looks like a squared tool with a groove in the middle, entirely metallic.*
